# Persistent Cognitive Dysfunction in a Non-Hospitalized COVID-19 Long-Hauler Patient Responding to Cognitive Rehabilitation and Citicoline Treatment

**DOI:** 10.3390/brainsci13091275

**Published:** 2023-09-01

**Authors:** Roberto Monastero, Roberta Baschi

**Affiliations:** Section of Neurology, Department of Biomedicine, Neuroscience and Advanced Diagnostics (BiND), University of Palermo, 90121 Palermo, Italy; roberta.baschi@gmail.com

**Keywords:** COVID-19, SARS-CoV-2, cognitive dysfunction, cognitive training, citicoline

## Abstract

The Severe Acute Respiratory Syndrome Coronavirus 2 (SARS-CoV-2) infection is characterized by severe flu-like symptoms, which can progress to life-threatening systemic inflammation and multiorgan dysfunction. The nervous system is involved in over one-third of patients, and the most common neurological manifestations concern the central nervous system, such as headache, fatigue, and brain fog. The activation of innate, humoral, and cellular immune responses, resulting in a cytokine storm and endothelial and mitochondrial dysfunctions, are the main pathophysiological mechanisms of SARS-CoV-2 infection. Citicoline is an exogenous source of choline and cytidine involved in intracellular phospholipid synthesis, which improves blood flow, brain activity, and mitochondrial dysfunction. This report will present the case of a non-hospitalized, 59-year-old female. After a mild form of SARS-CoV-2 infection, the patient developed cognitive disturbances such as forgetfulness and anomia. The multidimensional neuropsychological assessment revealed an impairment in episodic memory with borderline performance in executive and visuospatial functioning. Cognitive rehabilitation and treatment with citicoline 1000 mg/daily led to a marked improvement in symptoms after six months. Early identification of the neurological sequelae of the Coronavirus Disease 2019 (COVID-19) and timely rehabilitation interventions are required in non-hospitalized long-hauler patients with COVID-19. Long-term treatment with citicoline should be considered as potentially effective in improving cognitive functioning in subjects with Post COVID-19 Neurological Syndrome.

## 1. Introduction

The Coronavirus Disease 2019 (COVID-19), the causative agent of which is the Severe Acute Respiratory Syndrome Coronavirus 2 (SARS-CoV-2), spread worldwide in December 2019. The main symptoms affect the respiratory system, leading to severe respiratory failure [1]. However, neurological manifestations have also been described in over 35% of cases and subjects with mild and moderate cases of COVID-19 [2,3,4,5]. Multi-organ dysfunction may also persist in the post-acute phase of SARS-CoV-2 infection, constituting the so-called long COVID syndrome [1]. About 80% of patients have very limited and transient respiratory symptoms that do not require hospitalization; some patients, known as COVID-19 long-haulers, develop persistent and debilitating symptoms [6].

Understanding the pathophysiology of COVID-19 is crucial for exploring therapeutic options. SARS-CoV-2 is an RNA virus with a high affinity for angiotensin-converting enzyme 2 (ACE2) receptors expressed in various organs, including nervous tissue, vessels, and muscles [7]. Exposure to SARS-CoV-2 activates T cells through a mechanism related to Human Leukocyte Antigens (HLA) activation, inducing innate, humoral, and cellular immune responses. These mechanisms lead to a cytokine storm with activation of immune cells (macrophages, natural killer, T and B cells) [8]. Several proteins and enzymes are involved in the pathophysiological mechanisms of COVID-19, such as silent mating-type information regulation 2 homolog 1 (SIRT1) and phospholipase A2. In particular, the presence of mitochondrial dysfunction caused by SARS-CoV-2 infection may explain the brain fog reported by many patients with underlying infection, which is a hypoxic condition affecting neurons that require continuous oxygen supply at highly physiological levels [9].

The cytokine storm induced by SARS-CoV-2 infection causes endothelial dysfunction and alters blood–brain barrier (BBB) permeability [1]. The increased level of pro-inflammatory cytokines in the central nervous system (CNS) may persist even after recovery, thus contributing to possible long-term neuroinflammation. In the acute phase, neurological manifestations of COVID-19 infection involve, in order, the CNS (24.8%), skeletal muscle injury (10.7%), and the peripheral nervous system (8.9%) [2]. Persistent neurologic symptoms in non-hospitalized COVID-19 long-haulers have recently been described. Fatigue is the most reported symptom (more than 85%), followed by brain fog (81%), headache (68%), numbness/tingling (60%), dysgeusia (59%), and anosmia (55%) [6]. 

From a cognitive perspective, SARS-CoV-2-infected subjects showed worse performance in tasks evaluating attention and working memory, with mild-to-moderate cognitive dysfunction [6]. The latter cognitive deficits are present in patients without other residual symptoms of infection and are unaffected by differences in demographic or socioeconomic variables [10]. In the study of Zhou et al., patients aged 30 to 64 years who had recovered from COVID-19 showed worse performance in the domain of sustained attention and, interestingly, the latter impairment was positively correlated with the degree of inflammation [11]. Although some evidence has reported an association between cognitive impairment and the degree of pulmonary dysfunction and d-dimer levels during acute COVID-19 infection, no significant associations have been described with the length of hospitalization, total oxygen demand, or markers of severity such as lymphocytes, C-reactive protein, ferritin, or the highest recorded d-dimer level, although the latter appears to be correlated with delayed verbal recall and performance and psychomotor speed [12]. The estimated proportion of individuals with cognitive impairment 3 months after SARS-CoV-2 infection is more than 2%, according to a recent report by the Global Burden of Disease Long COVID Collaborators [13]. However, to date, there are no randomized clinical trials on therapeutic approaches for cognitive and neurological symptoms in patients with acute or prolonged COVID-19 infection.

Citicoline is a cognitive enhancer that acts on intracellular phospholipids synthesis and is an exogenous source of choline and cytidine [14]; in addition, the drug increases cerebral blood flow velocity and brain activity, improving cognitive performance [15]. The therapeutic effects of citicoline were described in a recent systematic review [16]. The latter study described that the drug showed the following significant beneficial effects: improvement in motor function, disability, and quality of life in patients with ischemic and hemorrhagic stroke; improvement in cognitive performance and improved prognosis and disability in patients with dementia of various etiologies. In contrast, in patients with traumatic brain injury, the efficacy of citicoline remains to be confirmed. In addition to the above-described effects, citicoline could potentially be used as a therapy for cases of cognitive decline related to COVID-19 because it has an anti-inflammatory action through inhibition of phospholipase 2 and upregulates the expression of SIRT1 protein levels in the brain, thus leading to improved neuronal plasticity and cognitive functioning [9]; furthermore, the drug has the potential to repair mitochondrial dysfunction and reduce oxidative damage [9,17]. Regarding rehabilitation techniques, memory training programs have been described as effective in subjects with cognitive decline and mild cognitive impairment [18]. There are no definitive data to date regarding cognitive rehabilitation in subjects with cognitive decline due to COVID-19 infection; however, a recent meta-analysis [19] suggests possible cognitive restorative approaches to avoid long-term cognitive complications from COVID-19. Herein, we describe the case of a 59-year-old, non-hospitalized woman with COVID-19 and persistent cognitive dysfunction who responded to citicoline and cognitive rehabilitation.

## 2. Case Presentation

SARS-CoV-2 infection was confirmed in early February 2021 in a 59-year-old right-handed female teacher by reverse transcriptase (RT)-PCR from a nasopharyngeal swab sample. The patient had no previous history of any disease nor a family history of neurodegenerative disease. The main symptoms were cough, fever, and dizziness. The patient had normal d-dimer values (77 ng/mL), and a chest Computed Tomography (CT) did not display the typical findings of COVID-19 pneumonia. The disease was very mild, and the patient self-isolated for approximately one month until a negative RT-PCR test was obtained. Subsequently, the patient started to complain of forgetfulness and anomia. Cognitive symptoms were slowly progressive and did not affect work performance or daily life.

In late April 2021, the patient was admitted to the Memory Clinic of the University Hospital of Palermo, where she performed a neurological assessment, a comprehensive diagnostic work-up and a multidimensional neuropsychological and behavioral assessment. A complete neurological examination was normal. The patient underwent a comprehensive blood biochemistry panel for COVID-19, including long COVID biomarkers (d-dimer, fibrinogen, albumin, ferritin, C-reactive protein, and interleukin 6) [20], and the parameters were all in the normal range. Lastly, 1.5 T brain Magnetic Resonance Imaging (MRI) with gadolinium revealed no significant vascular and/or parenchymal abnormalities, and the electroencephalogram (EEG) was normal.

Concerning neuropsychological assessment, the Mini-Mental State Examination [21] and the Montreal Cognitive Assessment [22] were administered as tests for global cognition. Functional abilities were evaluated with the Basic [23] (BADL) and the Instrumental [24] (IADL) Activities of Daily Living scales, respectively. Subjective Cognitive Impairment has been assessed through the Memory Assessment Clinics Questionnaire (MAC-Q) [25] (a six-item scale measuring subjective memory decline). 

The patient underwent a complete neuropsychological battery evaluating five cognitive domains: (1) working memory and short- and long-term memory, including Digit Span Forward (DSF) and Backward (DSB) [26], Rey Auditory Verbal Learning Test, Immediate [RAVLT-IR] and Delayed Recall [RAVLT-DR] [27], Story Recall Test [SR] [28]; (2) visuospatial and constructional functioning, including Clock Drawing [CD] [29] and Rey’s figure copy [30]; (3) selective and divided attention, including Trail Making Test A [TMT-A], Trial Making Test B [TMT-B] [31], and Attentive Matrices [28]; (4) executive functioning, including Raven’s Coloured Progressive Matrices [RPCM] [27] and Frontal Assessment Battery [FAB] [32]; language, including verbal [27] and semantic fluency [28] and the Token Test [TT] [28]. Raw scores were corrected for each test using Italian normative data for score adjustment (based on age, gender, and educational level) [28]. Anxiety and depression symptoms were assessed with the Hospital Anxiety and Depression Scale (HAD-A and HAD-D) [33], respectively. Table 1 shows the patient’s cognitive performance at baseline and after 6 months of follow-up. 

## 3. Results

Neuropsychological evaluation at baseline showed a deficit in verbal long-term episodic memory and borderline performance in executive functioning (inhibitory control and abstract reasoning) and visuo-constructional abilities. Specifically, an analysis of the RAVLT learning curve revealed a deficit of encoding, with consequent effects on learning and delayed recall. The RCPM demonstrated a slowdown in the processing of logical-operational skills, and the FAB revealed a loss of inhibitory control (i.e., scores 1/3 for the conflicting instructions and go-no-go subitems); the scores of both tests were slightly above the cut-off. Similarly, CD test performance was slightly impaired due to omissions and incorrect placement of the clock hands. Although performance on the verbal fluency test was within a normal range, several perseverative errors were recorded. Overall, the qualitative analysis of the above tests suggests a mild executive dysfunction. Performance in the remaining cognitive domains (attention, working memory, and language) was within the normal range. The HAD-A revealed a mild level of anxiety, probably related to the reported cognitive difficulties, while the HAD-D did not significantly show symptoms of depression. Lastly, the MAC-Q score revealed the presence of subjective memory impairment.

Based on the presence of a cognitive and mood disorder in a patient with previous COVID-19 infection, the patient was diagnosed with Post COVID-19 Neurological Syndrome (PCNS) [34] and treated with 1000 mg/daily oral citicoline, thereafter undergoing a cognitive rehabilitation treatment (two 40 min sessions per week, including relaxation techniques and mnemonic strategies inherent to verbal memory combined with spatial information retrieval with repeated presentations) for six months. A follow-up neuropsychological examination after six months revealed an overall improvement in cognitive performance, with a concomitant reduction in anxiety levels and the subjective reporting of memory deficits. As shown in Table 1, the patient achieved normal scores in all cognitive tests administered at follow-up, with only borderline performance in the delayed recall of the RAVLT, suggestive of residual impairment of verbal episodic memory.

## 4. Discussion

This study describes the case of a 59-year-old non-hospitalized woman with COVID-19 and persistent cognitive dysfunction, diagnosed as affected by PCNS [34], who responded to citicoline and cognitive rehabilitation. 

With reference to the pathophysiology of cognitive dysfunction in this patient, a possible rationale for the involvement of hypoxemia or vascular comorbidity [1] seemed unlikely, given that the subject did not experience respiratory symptoms requiring hospitalization, nor did she suffer from any vascular risk factors/diseases. Similarly, the activation of systemic inflammation associated with elevated serum levels of interleukin, resulting in increased permeability of the BBB [1,6], seems to be ruled out in this patient based on the normal values of acute-phase protein and IL-6 found 3 months after COVID-19 infection. In contrast, one possible mechanism that may have contributed to the neurological symptoms, in this case, includes the presence of brain damage from microvasculopathy, supported by megakaryocytes in the cortical capillaries of the brain, with the release of vasoactive substances such as serotonin [6]. Interestingly, citicoline was also found to be effective in acute ischemic stroke [16], including chronic subcortical hypoperfusion [35].

The patient was affected by COVID-19 with mild clinical manifestations, such as fever, cough, and dizziness. Complete blood biochemistry for long COVID was normal, and brain MRI and EEG revealed no significant morphological and electrical abnormalities. However, she started complaining of cognitive disturbances concerning verbal episodic memory, which was confirmed via formal neuropsychological testing. The latter also revealed a borderline impairment in executive functioning (inhibitory control and abstract reasoning) and visuospatial organization. Cognitive disturbances did not affect the patient’s ability to perform daily activities, including going to work.

The main cognitive domains involved in SARS-CoV-2 infection are attention and working memory [6], with a predominant frontoparietal hypometabolism [36]. In the case of our patient, verbal episodic memory was the most impaired cognitive function, along with mild dysfunction of executive functioning and visuospatial organization. These data suggest more diffuse brain involvement implicating the left mesial temporal cortex [37], in addition to the fronto-parietal involvement characteristic of executive [38] and visuospatial impairment [39], respectively.

An improvement in cognitive dysfunction was observed after a six-month combined rehabilitation program (with mnemonic and inhibitory control techniques) and the administration of 1000 mg/daily citicoline. The role of cognitive rehabilitation is well established in cognitive decline, particularly regarding memory functioning [18]; moreover, in our patient, the combination with citicoline therapy, a brain chemical known to improve brain activity [9,16], possibly led to further cognitive improvement. Citicoline has been shown to have anti-inflammatory (through the inhibition of phospholipase 2 activity), antiviral (being a proteasome regulator), and neuroprotective (reducing oxidative damage and improving mitochondrial dysfunction in the neocortex) properties [9]. In addition, citicoline serves as a substrate for synthesizing acetylcholine, a neurotransmitter essential for memory functioning and learning, and increases cerebral blood flow and velocity [16]. All these mechanisms could explain the beneficial effect of citicoline in our patients. Given the systemic involvement of the long COVID-19 syndrome, a multi-disciplinary approach to patients and specific, multi-organ rehabilitation programs are required. The latter also applies to long-hauler individuals requiring a prompt clinical assessment and related treatment.

## 5. Conclusions

To prevent long-term sequelae of COVID-19 and reduce subsequent disability in individuals with PCNS, the standardization of clinical assessments to stratify at-risk patients is required. In these patients, early rehabilitation programs and treatment with nootropic agents should be considered useful tools to prevent disability and improve the patient’s quality of life.

## Figures and Tables

**Table 1:** Patient’s performance on cognitive assessments at baseline and 6-month follow-up.

Tests	Baseline		6-Month Follow-Up		Cut-Off Point for Normal Performance
	Score	Classification	Score	Classification	
***Global cognition, functional ability, and subjective cognitive impairment***					
MMSE	26.31	normal	28.31	normal	>24
MOCA	23	*impaired*	27	normal	>25
ADL	6	normal	6	normal	-
IADL	8	normal	8	normal	-
MAC-Q	28	*impaired*	23	normal	<25
***Working memory, short and long-term memory***					
DSF	6.5	normal	7.5	normal	>3.75
DSB	3.58	normal	4.66	normal	>2.65
RAVLT-IR	27.2	*impaired*	31.2	normal	>28.53
RAVLT-DR	3	*impaired*	4	*impaired*	>4.69
Story Recall Test	9.5	normal	10.5	normal	>7.5
***Visuospatial and constructional functioning***					
Clock Drawing	7	*borderline*	11	normal	>6.54
Rey’s Figure copy	30	normal	33	normal	>28.87
***Selective and divided attention***					
Trail Making A	65	normal	63	normal	<94
Trail Making B	121	normal	111	normal	<283
Attentive Matrices	38	normal	40	normal	>31
***Executive functioning***					
RCPM	19.6	*borderline*	24.6	normal	>18.96
FAB	14.45	normal	16.45	normal	>12
***Language***					
Verbal fluency	28.6	normal	30	normal	>17.35
Semantic fluency	28	normal	30	normal	>24
Token Test	31.25	normal	32.25	normal	>26.5
***Behavioral Assessment***					
HAD-A	12	*impaired*	7	normal	<11
HAD-D	4	normal	3	normal	<11

Abbreviations: MMSE, Mini Mental State Examination; MOCA, Montreal Cognitive Assessment; ADL, Activities of Daily Living; IADL, Instrumental Activities of Daily Living; MAC-Q, Memory Assessment Clinics Questionnaire; DSF, Digit Span Forward; DSB, Digit Span Backward; RAVLT-IR and DR, Rey Auditory Verbal Learning Test, immediate recall and delayed recall; RCPM, Raven’s Coloured Progressive Matrices; FAB, Frontal Assessment Battery; HAD, Hospital Anxiety and Depression scale.

## Data Availability

The data presented in this study are available on request from the corresponding author. The data are not publicly available due to privacy reasons.

## References

[1] Crook H., Raza S., Nowell J., Young M., Edison P. (2021). Long covid—mechanisms, risk factors, and management. BMJ.

[2] Mao L., Jin H., Wang M., Hu Y., Chen S., He Q., Chang J., Hong C., Zhou Y., Wang D. (2020). Neurologic Manifestations of Hospitalized Patients with Coronavirus Disease 2019 in Wuhan, China. JAMA Neurol..

[3] Picone P., Sanfilippo T., Guggino R., Scalisi L., Monastero R., Baschi R., Mandalà V., San Biagio L., Rizzo M., Giacomazza D. (2022). Neurological Consequences, Mental Health, Physical Care, and Appropriate Nutrition in Long-COVID-19. Cell. Mol. Neurobiol..

[4] Torrente A., Alonge P., Di Stefano V., Baschi R., Ornello R., Correnti E., Lupica A., Camarda C., Farinella G., Raieli V. (2023). New-onset headache following COVID-19: An Italian multicentre case series. J. Neurol. Sci..

[5] Baschi R., Luca A., Nicoletti A., Caccamo M., Cicero C.E., D’Agate C., Di Giorgi L., La Bianca G., Lo Castro T., Zappia M. (2020). Changes in Motor, Cognitive, and Behavioral Symptoms in Parkinson’s Disease and Mild Cognitive Impairment During the COVID-19 Lockdown. Front. Psychiatry.

[6] Graham E.L., Clark J.R., Orban Z.S., Lim P.H., Szymanski A.L., Taylor C., DiBiase R.M., Jia D.T., Balabanov R., Ho S.U. (2021). Persistent neurologic symptoms and cognitive dysfunction in non-hospitalized Covid-19 “long haulers”. Ann. Clin. Transl. Neurol..

[7] Oudit G.Y., Wang K., Viveiros A., Kellner M.J., Penninger J.M. (2023). Angiotensin-converting enzyme 2—At the heart of the COVID-19 pandemic. Cell.

[8] Nicoli F., Solis-Soto M.T., Paudel D., Marconi P., Gavioli R., Appay V., Caputo A. (2020). Age-related decline of de novo T cell responsiveness as a cause of COVID-19 severity. GeroScience.

[9] Turana Y., Nathaniel M., Shen R., Ali S., Aparasu R.R. (2021). Citicoline and COVID-19-Related Cognitive and Other Neurologic Complications. Brain Sci..

[10] Hampshire A., Trender W., Chamberlain S.R., Jolly A.E., Grant J.E., Patrick F., Mazibuko N., Williams S.C.R., Barnby J.M., Hellyer P. (2021). Cognitive deficits in people who have recovered from COVID-19. eClinicalMedicine.

[11] Zhou H., Lu S., Chen J., Wei N., Wang D., Lyu H., Shi C., Hu S. (2020). The landscape of cognitive function in recovered COVID-19 patients. J. Psychiatr. Res..

[12] Miskowiak K., Johnsen S., Sattler S., Nielsen S., Kunalan K., Rungby J., Lapperre T., Porsberg C. (2021). Cognitive impairments four months after COVID-19 hospital discharge: Pattern, severity and association with illness variables. Eur. Neuropsychopharmacol..

[13] Wulf Hanson S., Abbafati C., Aerts J.G., Al-Aly Z., Ashbaugh C., Ballouz T., Blyuss O., Bobkova P., Bonsel G., Global Burden of Disease Long COVID Collaborators (2022). Estimated Global Proportions of Individuals With Persistent Fatigue, Cognitive, and Respiratory Symptom Clusters Following Symptomatic COVID-19 in 2020 and 2021. JAMA.

[14] Weiss S.B., Smith S.W., Kennedy E.P. (1958). The enzymatic formation of lecithin from cytidine diphosphate choline and d-1,2-diglyceride. J. Biol. Chem..

[15] Cacabelos R., Caamaño J., Gómez M.J., Fernández-Novoa L., Franco-Maside A., Alvarez X.A. (1996). Therapeutic Effects of CDP-Choline in Alzheimer’s Disease-Cognition, Brain Mapping, Cerebrovascular Hemodynamics, and Immune Factors. Ann. N. Y. Acad. Sci..

[16] Jasielski P., Piędel F., Piwek M., Rocka A., Petit V., Rejdak K. (2020). Application of Citicoline in Neurological Disorders: A Systematic Review. Nutrients.

[17] Stefano G.B., Ptacek R., Ptackova H., Martin A., Kream R.M. (2021). Selective Neuronal Mitochondrial Targeting in SARS-CoV-2 Infection Affects Cognitive Processes to Induce ‘Brain Fog’ and Results in Behavioral Changes That Favor Viral Survival. Med. Sci. Monit..

[18] Sherman D.S., Mauser J., Nuno M., Sherzai D. (2017). The Efficacy of Cognitive Intervention in Mild Cognitive Impairment (MCI): A Meta-Analysis of Outcomes on Neuropsychological Measures. Neuropsychol. Rev..

[19] Houben S., Bonnechère B. (2022). The Impact of COVID-19 Infection on Cognitive Function and the Implication for Rehabilitation: A Systematic Review and Meta-Analysis. Int. J. Environ. Res. Public Health.

[20] Lai Y.-J., Liu S.-H., Manachevakul S., Lee T.-A., Kuo C.-T., Bello D. (2023). Biomarkers in long COVID-19: A systematic review. Front. Med..

[21] Grigoletto F., Zappalà G., Anderson D.W., Lebowitz B.D. (1999). Norms for the Mini-Mental State Examination in a healthy population. Neurology.

[22] Nasreddine Z.S., Phillips N.A., Bedirian V., Charbonneau S., Whitehead V., Collin I., Cummings J.L., Chertkow H. (2005). The Montreal Cognitive Assessment, MoCA: A brief screening tool for mild cognitive impairment. J. Am. Geriatr. Soc..

[23] Katz S., Ford A.B., Moskowitz R.W., Jackson B.A., Jaffe M.W. (1963). Studies of illness in the aged. The index of ADL: A standardized measure of biological and psychosocial function. JAMA.

[24] Lawton M.P., Brody E.M. (1969). Assessment of older people: Self-maintaining and instrumental activities of daily living. Gerontologist.

[25] Crook T.H., Feher E.P., Larrabee G.J. (1992). Assessment of memory complaint in age-associated memory impairment: The MAC-Q. Int. Psychogeriatr..

[26] Monaco M., Costa A., Caltagirone C., Carlesimo G.A. (2013). Forward and backward span for verbal and visuo-spatial data: Standardization and normative data from an Italian adult population. Neurol. Sci..

[27] Carlesimo G.A., Caltagirone C., Gainotti G., Fadda L., Gallassi R., Lorusso S., Marfia G., Marra C., Nocentini U., Parnetti L. (1996). The Mental Deterioration Battery: Normative data, diagnostic reliability and qualitative analyses of cognitive impairment. Eur. Neurol..

[28] Spinnler H., Tognoni G. (1987). Standardizzazione e taratura italiana di test neuropsicologici. Ital. J. Neurol. Sci..

[29] Ricci M., Pigliautile M., D’Ambrosio V., Ercolani S., Bianchini C., Ruggiero C., Vanacore N., Mecocci P. (2016). The clock drawing test as a screening tool in mild cognitive impairment and very mild dementia: A new brief method of scoring and normative data in the elderly. Neurol. Sci..

[30] Caffarra P., Vezzadini G., Dieci F., Zonato F., Venneri A. (2002). Rey-Osterrieth complex figure: Normative values in an Italian population sample. Neurol. Sci..

[31] Giovagnoli A.R., Del Pesce M., Mascheroni S., Simoncelli M., Laiacona M., Capitani E. (1996). Trail making test: Normative values from 287 normal adult controls. Ital. J. Neurol. Sci..

[32] Appollonio I., Leone M., Isella V., Piamarta F., Consoli T., Villa M.L., Forapani E., Russo A., Nichelli P. (2005). The Frontal Assessment Battery (FAB): Normative values in an Italian population sample. Neurol. Sci..

[33] Zigmond A.S., Snaith R.P. (1983). The Hospital Anxiety and Depression Scale. Acta Psychiatr. Scand..

[34] Wijeratne T., Crewther S. (2020). Post-COVID 19 Neurological Syndrome (PCNS); a novel syndrome with challenges for the global neurology community. J. Neurol. Sci..

[35] Lee H.J., Kang J.S., Kim Y.I. (2009). Citicoline protects against cognitive impairment in a rat model of chronic cerebral hypoperfusion. J. Clin. Neurol..

[36] Hosp J.A., Dressing A., Blazhenets G., Bormann T., Rau A., Schwabenland M., Thurow J., Wagner D., Waller C., Niesen W.D. (2021). Cognitive impairment and altered cerebral glucose metabolism in the subacute stage of COVID-19. Brain.

[37] Cipolotti L., Bird C.M. (2006). Amnesia and the hippocampus. Curr. Opin. Neurol..

[38] Jones D.T., Graff-Radford J. (2021). Executive Dysfunction and the Prefrontal Cortex. Continuum.

[39] Supasitthumrong T., Herrmann N., Tunvirachaisakul C., Shulman K. (2019). Clock drawing and neuroanatomical correlates: A systematic review. Int. J. Geriatr. Psychiatry.

